# Causal analysis of the gut microbiota in differentiated thyroid carcinoma: a two-sample Mendelian randomization study

**DOI:** 10.3389/fgene.2023.1299930

**Published:** 2023-12-13

**Authors:** Zheng Quan, Xiaoyu Zhang, Shilong Wang, Yong Meng

**Affiliations:** ^1^ Department of Oncology Surgery, The Affiliated Hospital of Northwest University, Xi’an, China; ^2^ Department of Intensive Care Unit, The First Affiliated Hospital of Xi’an Jiaotong University, Xi’an, China; ^3^ Department of Surgical Oncology, The First Affiliated Hospital of Xi’an Jiaotong University, Xi’an, China

**Keywords:** gut microbiota, differentiated thyroid carcinoma, heterogeneity assessment, Mendelian randomization study, causal relationship

## Abstract

**Objective:** Numerous studies have highlighted an association between the gut microbiota (GM) and thyroid tumors. Employing Mendelian randomization methodology, we seek to elucidate the causal link between the gut microbiota and thyroid neoplasms.

**Methods:** We procured data from the Mibiogen database encompassing 211 distinct gut microbiota taxa, alongside extensive genome-wide association studies (GWAS) summary data for differentiated thyroid carcinoma (DTC). Our principal analytical approach involved the application of the Inverse-Variance Weighted method (IVW) within the framework of Mendelian randomization. Simultaneously, we conducted sensitivity analyses to assess result heterogeneity, horizontal pleiotropy, and outcome stability.

**Results:** IVW analysis revealed a dual role of the GM in thyroid carcinoma. The phylum Actinobacteria (OR, 0.249 [95% CI, 0.121–0.515]; *p* < 0.001) was associated with a decreased risk of DTC. Conversely, the genus Ruminiclostridium9 (OR, 11.276 [95% CI, 4.406–28.860]; *p* < 0.001), class Mollicutes (OR, 5.902 [95% CI, 1.768–19.699]; *p* = 0.004), genus RuminococcaceaeUCG004 (OR, 3.831 [95% CI, 1.516–9.683]; *p* = 0.005), genus Paraprevotella (OR, 3.536 [95% CI, 1.330–9.401]; *p* = 0.011), and phylum Tenericutes (OR, 5.902 [95% CI, 1.768–19.699]; *p* = 0.004) were associated with an increased risk of DTC.

**Conclusion:** Our findings underscore that the presence of genus Ruminiclostridium9, class Mollicutes, genus RuminococcaceaeUCG004, genus Paraprevotella, and phylum Tenericutes is associated with an elevated risk of DTC, whereas the presence of the phylum Actinobacteria is linked to a decreased risk. These discoveries enhance our comprehension of the relationship between the GM and DTC.

## 1 Introduction

Thyroid carcinoma, a common endocrine neoplasm of the head and neck, has experienced a steady increase in incidence, currently ranking as the fifth most prevalent cancer globally (Bray et al., 2018; WHO, 2020). Projections suggest that following its current trajectory, thyroid malignancies will become the fourth most common cancer in the United States by 2030 (Gonçalves et al., 2017). In 2020, global age-standardized incidence rates for thyroid cancer were 10.1 cases per 100,000 females and 3.1 cases per 100,000 males, with corresponding mortality rates of 0.5 and 0.3 cases per 100,000, respectively (Pizzato et al., 2022). An epidemiological survey covering 24% of the population in China (Yao et al., 2023) revealed that in 2019, the age-standardized incidence and mortality rates for thyroid cancer were 2.05 and 0.39 per 100,000, respectively. Over the last 30 years, the International Agency for Research on Cancer has noted an increasing incidence of thyroid cancer in diverse populations worldwide. In the United States, from 1970 to 2013, the annual growth rate of thyroid cancer incidence was reported to be 3% (Lim et al., 2017). Fortunately, the mortality rates for both males and females in most countries exhibit a stable or declining trend (Huang et al., 2023). DTC accounts for over 90% of all pathological diagnoses, with papillary carcinoma being its predominant histological subtype (Gu et al., 2014).The etiology of thyroid tumors is multifactorial, involving chromosomal mutations, genetic predisposition (Bonnefond and Davies, 2014), estrogen levels (Luo et al., 2016), ionizing radiation exposure (Bonnefond and Davies, 2014), autoimmune thyroid disorders (Khatami, 2009), and other factors. However, the risk factors for thyroid tumors are not fully understood, requiring additional research to uncover their pathogenic mechanisms.

Currently, the National Comprehensive Cancer Network (NCCN) guidelines recommend primary surgical intervention for DTC, reserving radioactive iodine-131 treatment for specific patient subsets (Haddad et al., 2022). Considering the global prevalence of thyroid tumors and their healthcare burden, as well as their profound impact on the wellbeing of affected individuals, our research is dedicated to uncovering the etiological underpinnings of this affliction.

Humans have coexisted with microorganisms throughout their existence, hosting a diverse array of microbes within various bodily niches, including the oral cavity, respiratory tract, gastrointestinal tract, genitourinary tract, and skin. Among these, the gut harbors the most intricate microbial ecosystem (Jiang et al., 2022). The human gut, in particular, teems with an assembly of microbial denizens numbering in the billions, with bacteria occupying the central stage (Gill et al., 2006). Such a vast consortium of GM also fulfills distinctive roles. Presently, microbiota are acknowledged for their substantial contributions to vitamin synthesis (B-complex vitamins, folate, vitamin K, among others) (Gu et al., 2016; Fang et al., 2017), facilitation of dietary fiber digestion, and regulation of immune responses (Bastiaanssen et al., 2019). Beyond these functions, microbiota also exhibit intricate associations with various diseases, encompassing gastrointestinal disorders, psychiatric illnesses, respiratory maladies, autoimmune conditions, and significantly, diverse malignancies, including lung, breast, colorectal, and esophageal cancers (Stebbings et al., 2002; Wang et al., 2016; Ishaq et al., 2021; Vitale et al., 2021).

A plethora of evidence has pointed toward the association between GM and thyroid malignancies, including thyroid carcinoma (Stebbings et al., 2002; Ishaq et al., 2021). (Ejtahed et al., 2020) Thyroid cancer patients exhibit dysbiosis in the gut microbiota, characterized by a reduction in the relative abundance of Faecalibacterium prausnitzii. Interestingly, an increase in the abundance of Faecalibacterium prausnitzii is observed after Radioactive Iodine Therapy (RAIT) (Fernandes et al., 2023). Lu et al. have identified significant alterations in the composition of the gut microbiota in thyroid cancer patients, with the *Bacteroides* enterotype emerging as the predominant bacterial type (Lu et al., 2022). Furthermore, gene sequencing results indicate a higher abundance of Firmicutes (Liu et al., 2021). In a study encompassing 74 patients, high-throughput sequencing was utilized to compare the microbial structural characteristics of 20 thyroid carcinoma patients, 18 thyroid nodule patients, and 36 healthy controls. The results underscored a close relationship between thyroid carcinoma, thyroid nodules, and altered microbiota (Zhang et al., 2019). Despite numerous indications suggesting an association between GM and thyroid malignancies, our understanding of this relationship remains incomplete, as these studies have not yet established causal links between thyroid tumors and GM.

Mendelian randomization represents an analytical framework harnessing genetic variation as instrumental variables (IV) to infer causal relationships between specific risk factors (i.e., exposures) and particular phenotypes (i.e., outcomes). Genetic variation, in this context, predominantly alludes to single nucleotide polymorphisms, signifying variations in specific nucleotides within the genetic material. In the realm of clinical investigation, myriad confounding factors often obscure the precision of our conclusions, rendering causal inferences tentative at best. The intrinsic merit of Mendelian randomization lies in its capacity to circumvent the impracticability of randomized controlled trials, such as the random allocation of microbiota to study individuals. Instead, this method leverages the natural grouping of single nucleotide polymorphisms (SNPs) and employs statistical techniques to ascertain the influence of SNPs on exposure and outcomes. nsequently, it helps to clarify causal associations between exposures and outcomes. Notably, owing to the even distribution of SNP loci, Mendelian randomization outcomes remain comparatively impervious to the interference of confounding factors, thereby conferring results akin to those derived from randomized controlled trials (Davies et al., 2018).

**FIGURE 1:**
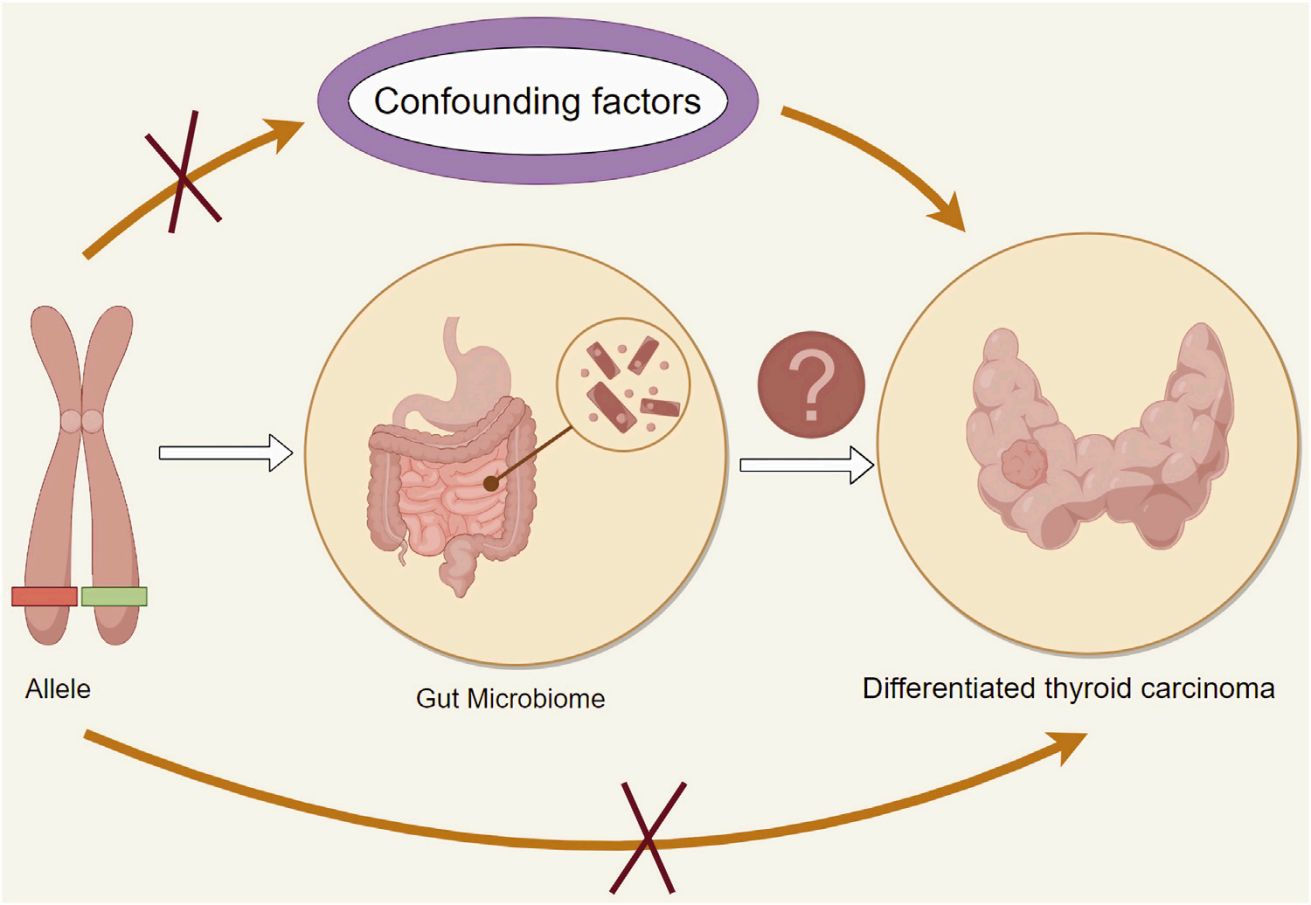
Study design concept and framework.

We utilize Mendelian randomization to disentangle the impact of confounding factors, enabling a precise evaluation of the causal relationship between GM and DTC., and Figure 1 provides an overview of the main research approach in this paper. This study’s overarching goal resides in employing Mendelian randomization as a methodological prism, utilizing genetic variation as instrumental variables to elucidate the causal nexus between microbiota and DTC. In doing so, we aspire to contribute novel evidence to the etiological and therapeutic paradigms within the domain of thyroid pathology.

## 2 Methods

### 2.1 Ethics statement

As the data used in this study comes from publicly available databases, after obtaining ethical approval from the Affiliated Hospital of Northwest University, Xi’an NO.3 hospital, the committee deemed formal ethical approval unnecessary. This decision was predicated upon the utilization of publicly accessible data devoid of identifiable patient information.

### 2.2 Study design

In this study, we define exposure as the GM, the outcome as malignant thyroid neoplasms, and instrumental variables as single nucleotide polymorphism (SNP) loci. In accordance with this premise, we discerned GM significantly associated with malignant thyroid neoplasms and subsequently proceeded with Mendelian randomization analysis. Throughout the course of MR analysis, we adhere to the following assumptions.

1. Associational Hypothesis: That is, SNP loci under investigation and GM demonstrate robust correlations. In our study, the significance threshold for the associational hypothesis is set at P < 1E-5.
2. Independence of SNPs and Confounding Factors: Among the SNPs ultimately incorporated into the MR study, those SNP loci exhibiting associations with either tumors or GM were excluded.
3. Exclusivity of Instrumental Variables’ Impact on Outcomes through Exposure: Instrumental variables should solely affect outcomes through the exposure and remain inert to other pathways, such as confounding. In essence, there should be no pleiotropy.

### 2.3 Data collection

The data pertaining to GM emanates from the consortium’s whole-genome association study summary data, Mibiogen. This dataset encapsulates 211 distinct taxonomic groups within the GM, spanning six taxonomic levels: kingdom, phylum, class, order, family, and genus. Access to this data can be procured from the website (https://mibiogen.gcc.rug.nl/). Notably, eight unidentified bacterial species were excluded from subsequent microbiota SNP locus analyses, leaving a total of 203 microbial SNPs for further investigation.

Thyroid tumor data, on the other hand, was sourced from Aleksandra Köhler et al.‘s prospective study (Köhler et al., 2013), which conducted a comprehensive genome-wide association study encompassing 701 patients afflicted with DTC. Diagnoses of thyroid tumors were ascertained through pathological results furnished by the Cisanello Hospital in Pisa, a prominent Italian referral center for thyroid disorders.

### 2.4 Variable selection

To assess the correlation between instrumental variables and microbiota, a filtration process for employed instrumental variables was enacted, involving the following steps:

1. The GM data, having been downloaded from the Mibiogen website, was validated and subsequently imported into R for analysis.
2. SNP loci exhibiting a stronger correlation with exposure were identified and filtered out based on a threshold of P < 1E-5 in the initial filtration process.
3. Instrumental variables demonstrating linkage disequilibrium were excluded. A standard of r2 > 0.001 and a physical distance (Kb) of 10,000 were employed for the removal of SNP loci exhibiting r^2^ values exceeding 0.001 with the most significant SNP within a 10,000 Kb range.
4. F-statistics were computed to assess the strength of instrumental variables. The calculation of F-statistics was predicated on beta values and standard errors (SE) for SNPs and exposure. In our study, all instrumental variables exhibited F-statistics exceeding 10.
5. SNP loci not conforming to the independence assumption were eliminated. We accessed the Phenoscanner database to identify secondary phenotypes associated with each SNP, verifying their correlation with confounding factors. SNP loci associated with both exposure outcomes were discarded based on criteria of *p*-value: < 1E-8 and r^2 > 0.8. Naturally, SNP loci directly correlated with thyroid tumors were also excluded. Following this comprehensive filtration, we gathered information regarding instrumental variables in outcomes and amalgamated effect sizes, commencing the MR analysis.

### 2.5 Statistical analyses

In the course of Mendelian randomization analysis, six distinct methodologies were employed, namely, IVW, IVW random-effects model, MR-Egger, MR-Egger bootstrap, Weighted Median, and Simple Median. Of these, the IVW analysis results, which calculated both the unadjusted *p*-values and the False Discovery Rate (FDR)-corrected *p*-values for each SNP locus, served as the primary analytical approach for this study. Multiple sensitivity analyses were additionally conducted, serving three primary objectives: firstly, to assess the robustness of the outcomes; secondly, to evaluate the potential presence of biases, including pleiotropy and data heterogeneity; and thirdly, to appraise the scenario where a specific instrumental variable unduly influenced the outcome.

To quantify heterogeneity in individual causal effects, Cochran’s Q was computed and subjected to examination, with a significance threshold of *p* ≤ 0.05 indicating the presence of pleiotropy. Within the context of heterogeneity testing, MR-Egger’s intercept and the Mendelian randomization residual sum and outlier (MR-PRESSO) method were employed. If the *p*-value exceeded 0.05, it indicated the absence of horizontal pleiotropy. All results underwent comprehensive visualization. To evaluate the scenario where a specific instrumental variable significantly impacted the outcome, a leave-one-out analysis was conducted by systematically excluding each SNP locus and observing the remaining SNPs’ Mendelian randomization. Finally, a reverse Mendelian randomization analysis was performed to ascertain the causal direction.

All statistical analyses were executed using the R programming language (https://www.r-project.org, R version 4.2.1). Statistical significance was deemed at *p* < 0.05. The initial date of analysis commenced in May 2023.

## 3 Results

### 3.1 Instrumental variable selection and initial MR results

Following our criteria, an initial set of 14,569 instrumental variable loci was established. Supplementary Table S1 provides a comprehensive breakdown of all microbiota details. By matching SNPs with thyroid tumor data, we obtained a subset of 3,302 SNPs.

Initial Mendelian randomization analysis yielded insights into the relationships between 203 GM and thyroid function, as presented in Supplementary Table S2. Based on the IVW-derived *p*-values, an initial selection identified 13 microbiota entities, namely,: Genus Ruminiclostridium 9 (ID:11357), Class Mollicutes (ID 3920), Phylum Tenericutes (ID:3919), Genus Ruminococcaceae UCG004 (ID:11362), Genus Paraprevotella (ID:962), Genus Ruminococcaceae UCG003 (ID:11361), Family Victivallaceae (ID:2,255), Genus Candidatus Soleaferrea (ID:11350), Phylum Actinobacteria (ID:400), However, through rigorous heterogeneity testing, horizontal pleiotropy assessments, and the exclusion of loci indirectly or directly related to thyroid tumor diseases, we ultimately distilled the selection down to six GM entities and 47 SNP loci as instrumental variables: Genus Ruminiclostridium 9 (ID:11357), Class Mollicutes (ID:3920), Genus Ruminococcaceae UCG004 (ID:11362), Genus Paraprevotella (ID:962), Phylum Actinobacteria (ID:400), Phylum Tenericutes (ID:3919).

The secondary features of these aforementioned SNPs were queried using PhenoScanner and are documented in Supplementary Table S3. It is noteworthy that these features have been confirmed as non-pleiotropic factors contributing to thyroid tumor etiology.

### 3.2 Detailed Mendelian randomization analysis results

**FIGURE 2:**
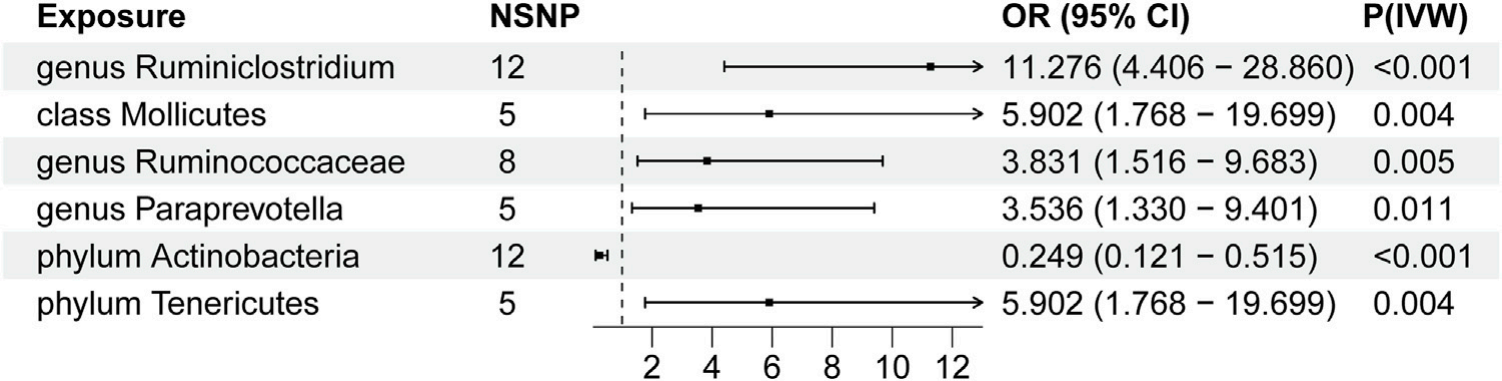
Forest Plot of Primary MR Analysis Results. NSNP = number of SNPs; OR = odds ratio; P = significance *p*-value; IVW = Inverse-Variance Weighted method.

We conducted Mendelian randomization analysis on the final set of six GM and 47 SNP loci. IVW results revealed significant associations as follows: genus Ruminiclostridium9(OR, 11.276, [95% CI, 4.406–28.860 ]; *p* < 0.001), class Mollicutes (OR, 5.902, [95% CI, 1.768–19.699]; *p* = 0.004), genus RuminococcaceaeUCG004(OR, 3.831, [95% CI, 1.516–9.683 ]; *p* = 0.005), genus Paraprevotella (OR, 3.536, [95% CI, 1.330–9.401 ]; *p* = 0.011), phylum Tenericutes (OR, 5.902 [95% CI, 1.768–19.699 ]; *p* = 0.004)exhibited an elevated risk of thyroid tumors, whereas phylum actinobacteria (OR, 0.249 [95% CI, 0.121–0.515]; *p* < 0.001)demonstrated a decreased risk of thyroid tumors. These findings are graphically depicted in the forest plot (Figure 2). Additional MR analysis outcomes are presented in Supplementary Table S4. In our findings, IVW and IVW-MRE methods demonstrated consistency, while other methodologies may yield differing results compared to IVW and IVW-MRE. We posit that the IVW approach derives an overall effect by weight-averaging estimates across distinct loci, whereas IVW-MRE additionally accounts for measurement error, employing a random-effects model to estimate the total effect. Discrepancies among other methods may be attributed to sample size, skewness, or heterogeneity. In case of inconsistencies, pay particular attention to the results from IVW and IVW-MRE methods, considering the unique attributes of alternative methodologies.

**FIGURE 3:**
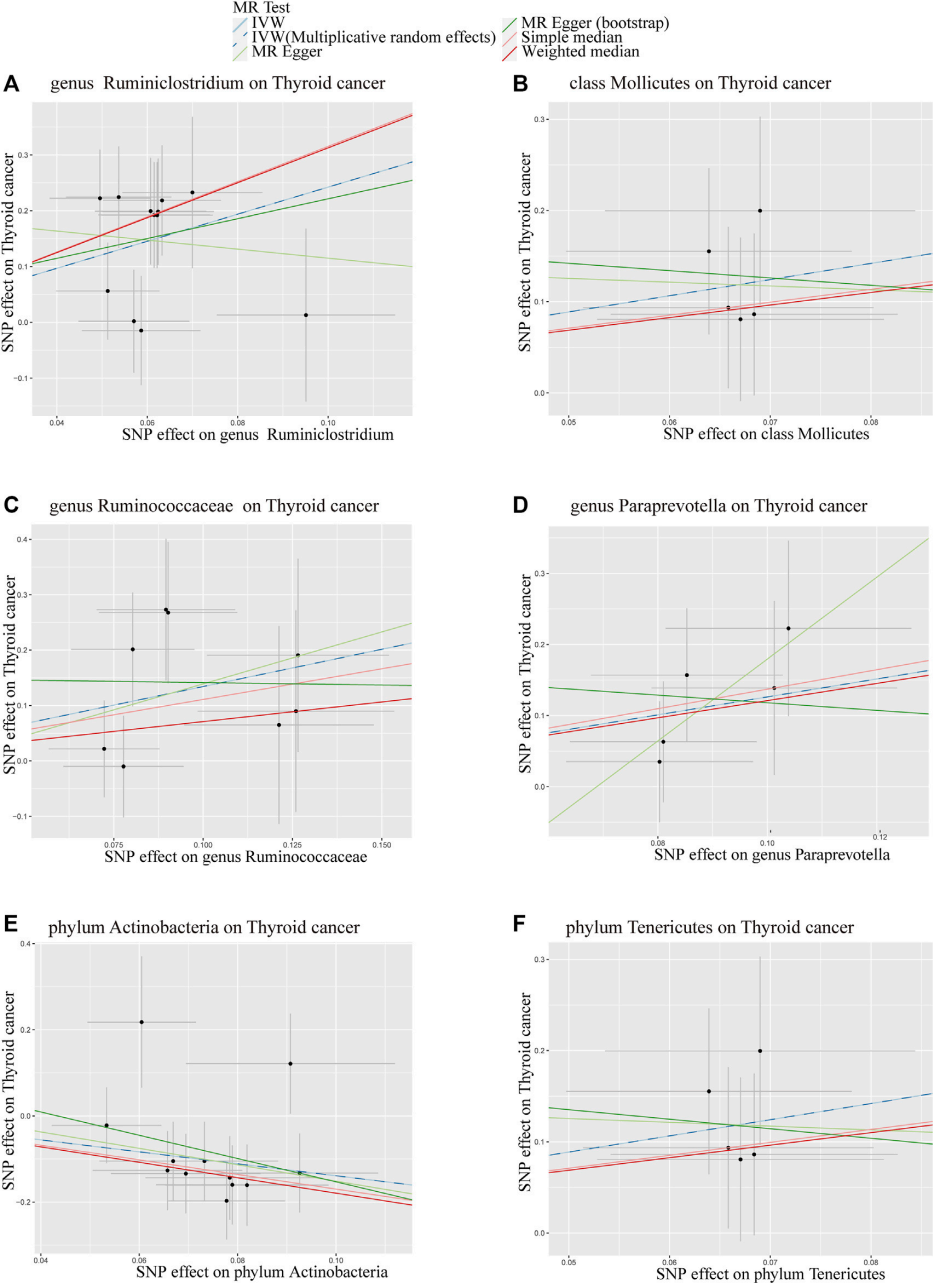
Scatter Plot of MR Analysis for the Influence of 6 Gut Microbiota on Thyroid Tumors. **(A)** = genus Ruminiclostridium; **(B)** = class Mollicutes; **(C)** = genus Ruminococcaceae; **(D)** = genus Paraprevotella; **(E)** = phylum Actinobacteria; **(F)** = phylum Tenericutes; MR Test = Statistical analysis methods.

To visualize our findings comprehensively, we presented all results in a scatter plot (Figure 3), where each data point represents an SNP, the upper and lower lines delineate confidence intervals, and the horizontal and vertical axes respectively denote the SNP’s effects on GM and thyroid tumor outcomes. The colored lines signify the fitting effects of the MR. Intriguingly, IVW and IVW-MRE methods exhibited remarkable consistency across all results.

### 3.3 Sensitivity analysis

Initially, we conducted heterogeneity checks on the results obtained from the selected six bacteria and 47 SNP loci. We observed that all I2 values for the microbiota were <50%, and the *p*-values obtained using two different methods were both greater than 0.05 (Table 1). This suggests that our results exhibited minimal heterogeneity. Additionally, the MR-PRESSO outlier test did not identify any anomalies (Table 1). To assess the horizontal pleiotropy of SNP loci, we utilized the global test from MRPRESSO and the MR-Egger intercept test. The *p*-values for all tests of horizontal pleiotropy exceeded 0.3, signifying that the impact of instrumental variables on thyroid cancer is unlikely to be influenced by factors other than the microbiota (Table 1).

**TABLE 1:** Heterogeneity and horizontal pleiotropy test results for gut microbiota.

Gut microbes	Heterogeneity test				MR-PRESSO				MR-Egger	
	IVW	*p*-value	MR-Egger	P	Global test	P	Outlier-corrected	P	Egger intercept test	P
Genus Ruminiclostridium	11.357	0.414	10.404	0.406	1.795	0.904	NA	NA	0.196	0.361
Class Mollicutes	1.180	0.881	1.171	0.760	1.794	0.892	NA	NA	0.146	0.930
Genus Ruminococcaceae	6.376	0.497	6.324	0.388	12.336	0.517	NA	NA	−0.048	0.831
Genus Paraprevotella	1.656	0.799	0.762	0.859	13.459	0.474	NA	NA	−0.396	0.414
Phylum Actinobacteria	10.804	0.460	10.761	0.376	8.457	0.501	NA	NA	0.039	0.846
Phylum Tenericutes	1.180	0.881	1.171	0.760	2.621	0.82	NA	NA	0.146	0.930

Heterogeneity tests were conducted using Cochran Q for both IVW, and MR-Egger, while horizontal pleiotropy was assessed using MR-PRESSO, and the Egger intercept test.

**FIGURE 4:**
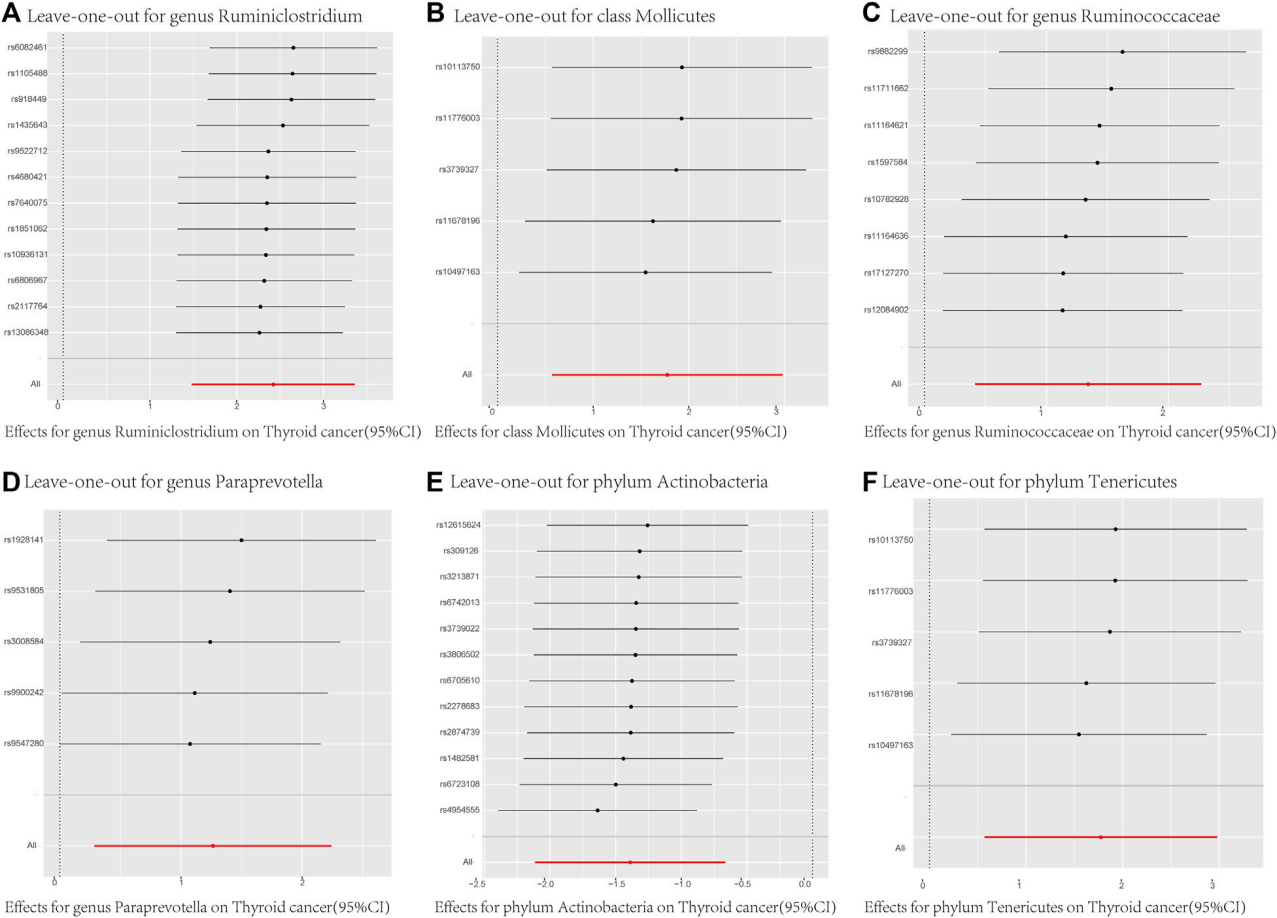
MR Analysis Results Using Leave-One-Out for Different SNP Loci of the 6 Gut Microbiota. **(A)** = genus Ruminiclostridium; **(B)** = class Mollicutes; **(C)** = genus Ruminococcaceae; **(D)** = genus Paraprevotella; **(E)** = phylum Actinobacteria; **(F)** = phylum Tenericutes; MR Test = Statistical analysis methods.

In addition to MR-Egger and MR-PRESSO, we conducted individual SNP MR estimates (Supplementary Figure S1) and systematically removed individual SNPs to compute the remaining SNPs’ Mendelian randomization effects (Figure 4). Leave-one-out analysis, where each SNP is excluded in turn, showed that all error bars were consistently on the right or left of zero in Figure 4. This indicates minimal variation in the overall error bars, signifying high robustness in the results. The causal estimates were not driven by any single SNP. In the forest plot of Supplementary Figure S1, each solid horizontal line represents the results estimated using the Wald ratio method for individual SNPs, while the red line represents the composite outcome, reflecting the risk of thyroid cancer for each microbiota under the IVW method.

### 3.4 Reverse MR analysis

In the reverse MR analysis, exposure and outcome were interchanged. However, we did not observe any significant causal relationship between the outcome and exposure, except in the case of the Phylum Tenericutes using the MR Egger method, where the *p*-value exceeded 0.05 (Table 2).

**TABLE 2:** Reverse MR analysis of the main results.

Outcome	method	NSNP	b	se	*p*-value	*p*-value (MR-PRESSO)
Phylum Tenericutes	Inverse variance weighted	328	−0.000413	0.0006304	0.512	0.561
	MR Egger	328	−0.002639	0.0011036	0.017	
	Simple mode	328	−0.001691	0.0019129	0.377	
	Weighted median	328	−0.000413	0.0009814	0.674	
	Weighted mode	328	0.0002101	0.0013032	0.872	
Phylum Actinobacteria	Inverse variance weighted	329	0.0003186	0.0005009	0.525	0.977
	MR Egger	329	2.73E-05	0.0008769	0.975	
	Simple mode	329	−0.001726	0.00158	0.275	
	Weighted median	329	−0.000538	0.0007755	0.488	
	Weighted mode	329	−0.001229	0.0011322	0.278	
Genus Ruminococcaceae	Inverse variance weighted	328	−0.000573	0.0006887	0.405	0.751
	MR Egger	328	0.0008787	0.0012057	0.467	
	Simple mode	328	0.0003512	0.0020584	0.865	
	Weighted median	328	−3.73E-05	0.0010136	0.971	
	Weighted mode	328	0.0007602	0.0014312	0.596	
Genus Ruminiclostridium	Inverse variance weighted	329	0.0002747	0.0005414	0.612	0.126
	MR Egger	329	0.0009264	0.0009481	0.329	
	Simple mode	329	−0.000906	0.0016819	0.591	
	Weighted median	329	0.0004644	0.0007995	0.561	
	Weighted mode	329	0.00038	0.0010821	0.726	
Genus Paraprevotella	Inverse variance weighted	328	−0.000161	0.0007957	0.840	0.781
	MR Egger	328	0.0004046	0.0013927	0.772	
	Simple mode	328	0.001209	0.0021467	0.574	
	Weighted median	328	−0.000415	0.0011592	0.720	
	Weighted mode	328	−0.000828	0.001428	0.563	
Class Mollicutes	Inverse variance weighted	328	−0.000413	0.0006304	0.512	0.555
	MR Egger	328	−0.002639	0.0011036	0.017	
	Simple mode	328	−0.001691	0.0016845	0.316	
	Weighted median	328	−0.000413	0.0009433	0.661	
	Weighted mode	328	0.0002101	0.001257	0.867	

NSNP = number of SNPs; b = effect size; se = standard error; pval = *p*-value.

We conducted a global test for horizontal pleiotropy using the MR-PRESSO method, and no evidence of horizontal pleiotropy was detected among the SNPs. Moreover, no outliers were detected in the outlier test. Hence, we have reasonable grounds to posit that these various bacteria are causative factors for thyroid tumors, rather than being outcomes of the condition.

## 4 Discussion

In spite of the prior research on the relationship between the GM and thyroid cancer, the concept of the association between DTC and the GM remains relatively uncharted (Samimi and Haghpanah, 2020). To the best of our knowledge, this study represents the first causal investigation into the link between the GM and DTC. Through a Mendelian randomization analysis involving two-sample datasets, we report that five microbiota entities, namely, genus Ruminiclostridium 9 (*p* < 0.001), class Mollicutes (*p* = 0.004), genus Ruminococcaceae UCG004 (*p* = 0.005), genus Paraprevotella (*p* = 0.011), and phylum Tenericutes (*p* = 0.004), are associated with an increased risk of developing non-differentiated thyroid carcinoma. Additionally, phylum Actinobacteria (*p* < 0.001) appears to be associated with a lower the risk of non-differentiated thyroid carcinoma. We utilized various sensitivity analysis techniques to affirm the reliability and robustness of our findings.

The GM is known to be influenced by a multitude of factors. Notably, infants born via cesarean section exhibit lower diversity in their GM(31). Throughout one’s life, the GM remains under the continual influence of various factors including diet, medication, genetics, environment, disease, and the use of antibiotics (Maslowski and Mackay, 2011). This substantiates the significance of the GM as a potential therapeutic target for diseases.

Most of the research concerning the GM has been concentrated on gastrointestinal diseases, such as colorectal cancer and inflammatory bowel disease. In the context of colorectal cancer, gut bacteria may promote tumorigenesis by influencing bile acid secretion or undergoing changes in their taxonomic composition. Decreased bile acid secretion results in gut dysbiosis, accelerating inflammation and DNA damage, thereby directly contributing to tumorigenesis (Louis et al., 2014; Ridlon et al., 2014). Furthermore, an increase in *Fusobacterium* nucleatum, a type of gut bacteria, can promote colon tumorigenesis through its metabolites and cytotoxicity, (Wu et al., 2004; Wu et al., 2009), thereby affecting signaling pathways such as E-cadherin, NF-κB, and STAT3 (Maslowski and Mackay, 2011; Rautava et al., 2012). These studies suggest that the role of microbiota in tumorigenesis seems to be associated with DNA damage and the regulation of local inflammation via metabolic products.

Microbes can impact thyroid disease through various pathways. The GM may influence the secretion of thyroid-stimulating hormones via the hypothalamus-pituitary axis, thereby playing a role in thyroid diseases (Fröhlich and Wahl, 2019). For instance, in studies of patients with Hashimoto’s thyroiditis (HT), a form of hypothyroidism, researchers observed dysbiosis in the patients’ GM, along with overgrowth of certain microbes (Ishaq et al., 2017; Zhao et al., 2018). This influence is thought to occur because thyroid-related nutrients need to be acquired through the gut (Knezevic et al., 2020). Furthermore, in Graves’ disease patients, there is a higher abundance of Bacteroidaceae and Prevotellaceae and a lower abundance of Veillonellaceae, Enterobacteriaceae, and Lachnospiraceae compared to healthy individuals (Ishaq et al., 2018). In the realm of researching the relationship between the GM and thyroid cancer, the first study proposing a potential connection between thyroid cancer and the GM was published in 2017. Shen et al. employed gas chromatography-time-of-flight mass spectrometry to analyze the serum of thyroid cancer patients with and without distant metastases. They found elevated levels of serum ammonia, pyruvic acid, and γ-aminobutyric acid in patients with distant metastases, suggesting a potential connection to differences in GM or diet (Shen et al., 2017). The first study to specifically investigate the relationship between thyroid cancer and the GM was conducted by Jing Feng in 2019 (Feng et al., 2019). Feng reported higher gut microbial richness and α-diversity in patients with TC compared to healthy controls, but the sample size was limited (30 patients from Harbin, China, *versus* 35 healthy controls), and the study design was observational. In other observational studies, GM taxa that were found to be increased in abundance in thyroid cancer patients included Clostridiaceae, Nesterenkonia, and *Streptococcus* (Zhang et al., 2019), while decreased taxa primarily included *Lactobacillus* (Zhang et al., 2019), *Bacteroides*, *Clostridium*, and Prevotella. These studies shed light on the potential link between the GM and thyroid cancer.

Our study highlights the significance of five specific microbiota entities—genus Ruminiclostridium 9, class Mollicutes, genus Ruminococcaceae UCG004, genus Paraprevotella, phylum Tenericutes, and phylum Actinobacteria—in relation to DTC.

Genus Ruminiclostridium 9, formerly known as Ruminoclostridium, is a characteristic bacterium found in the colon. Research indicates that interactions among colonic microorganisms are more intricate compared to the duodenum (Wu et al., 2020). This bacterium is associated with various diseases; it can reduce the risk of Alzheimer’s disease (OR 0.969, 95% CI 0.943–0.996, *p* = 0.009) (Ning et al., 2022). Furthermore, it has a positive correlation with cognitive function (Guo et al., 2021). In tumor immunology studies, Ruminiclostridium has shown a negative correlation with CD8^+^ T cells (Singh et al., 2023). In an autoimmune model of the central nervous system, medium-chain fatty acids (MCFAs) produced by this genus counteract the anti-inflammatory effects of short-chain fatty acids (SCFAs) by enhancing TH1 and TH17 cell differentiation (Haghikia et al., 2015).Therefore, certain genera, such as Ruminiclostridium 9, may contribute to the initiation of DTC through analogous mechanisms, potentially by reducing short-chain fatty acidsand modulating the immune response.

Class Mollicutes, representing the smallest self-replicating bacteria without cell walls, belongs to the phylum Tenericutes (Chernova et al., 2021). Mollicutes exhibit versatility, as they have been associated with both decreased relative abundance in severe depression patients (Zhu et al., 2019) and as a risk factor for Graves’ disease (Cao et al., 2023). Phylum Tenericutes has also been linked to various diseases, such as breast cancer (Niccolai et al., 2023), Crohn’s disease (Russo et al., 2022), lower risk of intrahepatic cholestasis of pregnancy (Li et al., 2023), and polycystic ovary syndrome (Li et al., 2023). However, research on the mechanisms underlying these associations remains limited. Genus Ruminococcaceae, like Ruminiclostridium, is present in the colonic mucosa. A decrease in Ruminococcaceae has been associated with various inflammatory bowel diseases, including ulcerative colitis and Crohn’s disease (Sokol et al., 2008; Joossens et al., 2011; Morgan et al., 2012). This bacterium produces short-chain fatty acids (SCFAs) and other small molecules, which serve as an energy source for colonic epithelial cells. A deficiency in these SCFAs may lead to disturbances and dysfunction in colonic mucosa (Young and Schmidt, 2004; Wong et al., 2006). SCFAs, particularly butyrate, are known to influence immune regulation and possess anti-inflammatory properties (Köhling et al., 2017), and butyrate can inhibit the activity and life cycle of cancer cells (Davie, 2003). This knowledge, seemingly at odds with our study results, suggests the involvement of unknown mechanisms. Interestingly, it has also been identified as a potential etiological factor contributing to Graves’ Disease (GD) (Cao et al., 2023). This association may be attributed to the ability of short-chain fatty acids (SCFA) to inhibit histone deacetylases (HDAC) and activate the re-expression of transport proteins in thyroid cancer cells, thereby inducing the differentiation of tumor cells and enhancing iodine uptake (Zhou et al., 2018; Rathod et al., 2020). Genus Paraprevotella has been the subject of several studies. It is a polymorphic, anaerobic, non-spore-forming Gram-negative rod isolated from human feces (Morotomi et al., 2009). This bacterium exhibits complex effects. On one hand, it is more abundant in individuals with genetic longevity (Liu et al., 2023) and correlates with small intestinal mucosal healing in Crohn’s disease patients (Hattori et al., 2020). On the other hand, it is more abundant in patients with heart failure and depression (Gutiérrez-Calabrés et al., 2020) and positively correlates with the severity of depression (Liśkiewicz et al., 2021). Previous studies have indicated its utility in distinguishing untreated primary hypothyroidism patients from healthy individuals.Currently, Paraprevotella is considered to have a positive correlation with plasma butyrate and valerate concentrations and contributes to the regulation of colonic motility, possibly through regulating fecal butyrate levels and serum IL-8 concentrations. The Paraprevotella strain proves to be an efficient pancreatic protease-degrading symbiont. The autolysis of pancreatic proteases facilitates bacterial invasion and destruction, potentially culminating in inflammation and injury, thereby creating a conducive environment for the onset of thyroid cancer.In summary, Paraprevotella is associated with various diseases, but its potential impact on human health remains unclear (Morotomi et al., 2009), and its specific mechanisms are yet to be explored. Phylum Actinobacteria is one of the most diverse bacterial phyla in nature (Lewin et al., 2016). Due to its diversity, it has both positive and negative effects on human health. Actinobacteria includes many bacteria that produce antibiotics, such as *Streptomyces*, which is known for synthesizing eptomycin, kanamycin, chloramphenicol, and erythromycin (Bérdy, 2005). It also encompasses several human health-threatening pathogens, such as *Mycobacterium tuberculosis*, which causes pulmonary tuberculosis. In the order Bifidobacteriales, members like Bifidobacterium are known for their beneficial effects on host health. A lower abundance of these bacteria has been associated with various diseases (Binda et al., 2018). Some researchers propose that species of Bifidobacterium in the human gut may contribute to host health by exerting antibacterial activity against pathogens and possibly by aiding in the development and function of the immune system as defensive symbionts (Servin, 2004). This aligns with our research findings. We also postulate that Actinobacteria may indirectly inhibit the occurrence of thyroid cancer by suppressing the processes of inflammation and oxidative stress.

What mechanisms might bacteria employ to induce DTC? Carcinogenesis primarily relies on two mechanisms: DNA damage and cell apoptosis, as well as the immune surveillance against tumor growth (Docimo et al., 2020; Liu et al., 2022). Intestinal bacteria can impact tumor proliferation through both of these mechanisms. New mechanisms involving bacteria and their metabolites or toxins causing direct DNA damage and carcinogenic mutations have been identified. For instance, infection with *Enterococcus* can lead to an increase in hydroxyl free radical production, resulting in DNA damage (Wang and Huycke, 2007). Additionally, oxidative stress can disrupt the homeostasis of the host gut microbiota (Riaz Rajoka et al., 2021). Therefore, drugs targeting both oxidative stress and gut microbiota may hold prognostic and therapeutic significance for thyroid cancer.Concerning immune surveillance, the gut microbiota significantly regulates the balance within the body and the development of immune cells. It modulates both innate and adaptive immune systems, particularly outside the intestinal tract (Maslowski and Mackay, 2011), making it a potential immune modulator (Pitt et al., 2016). As it is widely known, more than 70% of the entire immune system is associated with the gastrointestinal lymphoid tissues, and the gut microbiota can regulate immune balance and cell development (Maslowski and Mackay, 2011; Docimo et al., 2020). Certain metabolic products can also induce autoimmune reactions, leading to an imbalance in endocrine homeostasis and the occurrence of autoimmune diseases (Liu et al., 2022). Microbial dysbiosis can stimulate CD8 (+) T cells to promote chronic inflammation and early T-cell exhaustion, thereby diminishing the immune capacity against tumors (Yu et al., 2020). Therefore, the gut microbiota holds significant potential as a biomarker for predicting immune-related adverse events (Von Itzstein et al., 2020) and may influence the progression of thyroid cancer through immune modulation.In the future, we can develop novel strategies for diagnosing, predicting prognosis, actively monitoring, and intervening in DTC by studying the correlation between different bacterial enterotypes and thyroid cancer.This study also presents certain limitations. Firstly, we solely analyzed common microbiota and SNP loci, leaving unidentified microbiota and SNPs unexamined, thereby limiting our analysis. Secondly, our GWAS data was derived from individuals of European descent, and we did not identify other patient characteristics, which may restrict the generalizability of our conclusions to other populations, given the varying compositions of microbiota across different countries and ethnicities. Additionally, while we employed different SNP loci as “natural groupings,” it is essential to acknowledge the numerous unknown or potential interactions among distinct SNP loci. Finally, it is essential to note that this study is an observational investigation, lacking foundational research to mechanistically substantiate the observed outcomes. In the future, in addition to requiring more Genome-Wide Association Studies (GWAS) and microbiota data, further in-depth research is warranted to elucidate the mechanisms underlying the association between gut microbiota and thyroid tumor development. These studies will contribute to enhancing our understanding of the causal link between thyroid cancer and the gut microbiota.

In conclusion, our findings indicate a bidirectional role of GM in thyroid cancer. Genus Ruminiclostridium9, class Mollicutes, genus RuminococcaceaeUCG004, genus Paraprevotella, and phylum Tenericutes were associated with an increased risk of undifferentiated thyroid cancer, while phylum Actinobacteria (*p* < 0.001) was associated with a reduced risk of undifferentiated thyroid cancer. However, the underlying processes involved are intricate, necessitating further mechanistic research.

## Data Availability

The original contributions presented in the study are included in the article/Supplementary Material, further inquiries can be directed to the corresponding author.
