# Supplementary material for: Causal analysis of the gut microbiota in differentiated thyroid carcinoma: a two-sample Mendelian randomization study
Source: Front Genet. 2023 Dec 13;14:1299930. doi: 10.3389/fgene.2023.1299930 (PMC10753834; doi:10.3389/fgene.2023.1299930)
Supplement: Supplementary file 3 [file DataSheet1.PDF]

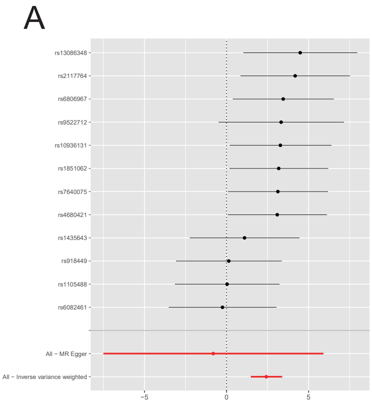


MR Effects for genus Ruminiclostridium on Thyroid cancer(95%CI)

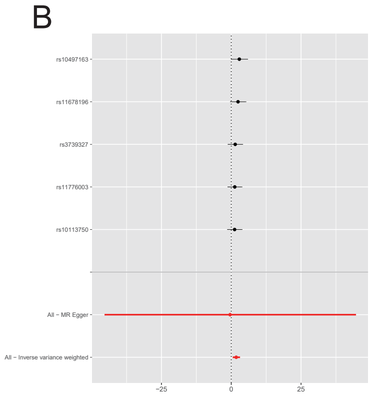


MR Effects for class Mollicutes on Thyroid cancer(95%CI)

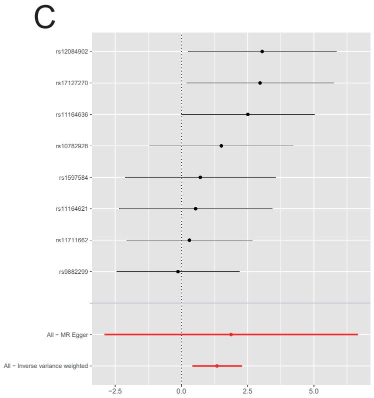


MR Effects for genus Ruminococcaceae on Thyroid cancer(95%CI)

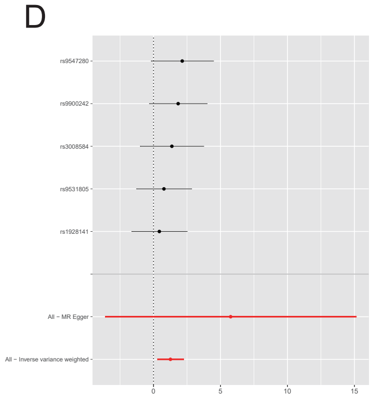


MR Effects for genus Paraprevotella on Thyroid cancer(95%CI)

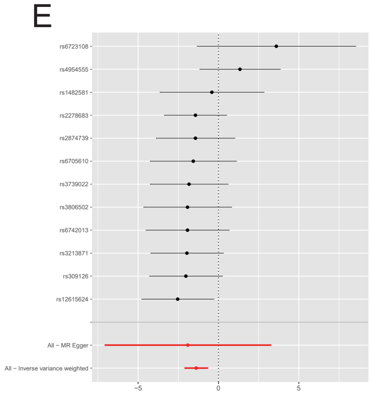


MR Effects for phylum Actinobacteria on Thyroid cancer(95%CI)

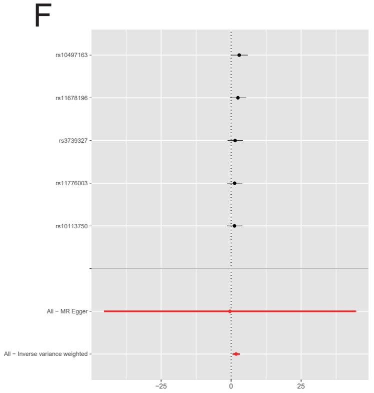


MR Effects for phylum Tenericutes on Thyroid cancer(95%CI)
