# Supplementary material for: Causal analysis of the gut microbiota in differentiated thyroid carcinoma: a two-sample Mendelian randomization study
Source: Front Genet. 2023 Dec 13;14:1299930. doi: 10.3389/fgene.2023.1299930 (PMC10753834; doi:10.3389/fgene.2023.1299930)
Supplement: Supplementary file 5 [file Table4.DOCX]

Supplement Table 4. Major Results Using Six MR Analysis Methods

| Outcome | Exposure | method | NSNP | b | se | pval | OR | Lower 95%CI | Upper 95%CI | FDR |
| --- | --- | --- | --- | --- | --- | --- | --- | --- | --- | --- |
| Thyroid cancer | genus Ruminiclostridium | Inverse variance weighted | 12 | 2.423 | 0.479 | <0.001 | 11.276 | 4.406 | 28.860 | 3.92E-06 |
| Thyroid cancer | genus Ruminiclostridium | Inverse variance weighted (multiplicative random effects) | 12 | 2.423 | 0.479 | <0.001 | 11.276 | 4.406 | 28.860 | 3.92E-06 |
| Thyroid cancer | genus Ruminiclostridium | MR Egger | 12 | -0.810 | 3.412 | 0.817 | 0.445 | 5.54E-04 | 3.57E+02 | 0.865 |
| Thyroid cancer | genus Ruminiclostridium | Simple median | 12 | 3.154 | 0.661 | <0.001 | 23.423 | 6.418 | 85.486 | 1.30E-05 |
| Thyroid cancer | genus Ruminiclostridium | MR Egger (bootstrap) | 12 | 1.863 | 2.367 | 0.213 | 6.444 | 0.062 | 6.67E+02 | 0.307 |
| Thyroid cancer | genus Ruminiclostridium | Weighted median | 12 | 3.127 | 0.705 | <0.001 | 22.801 | 5.729 | 90.749 | 5.48E-05 |
| Thyroid cancer | class Mollicutes | Inverse variance weighted | 5 | 1.775 | 0.615 | 0.004 | 5.902 | 1.768 | 19.699 | 0.010 |
| Thyroid cancer | class Mollicutes | Inverse variance weighted (multiplicative random effects) | 5 | 1.775 | 0.334 | <0.001 | 5.902 | 3.067 | 11.357 | <0.001 |
| Thyroid cancer | class Mollicutes | MR Egger | 5 | -0.417 | 22.924 | 0.987 | 0.659 | 2.02E-20 | 2.15E+19 | 0.987 |
| Thyroid cancer | class Mollicutes | Simple median | 5 | 1.420 | 0.831 | 0.087 | 4.137 | 0.812 | 21.085 | 0.137 |
| Thyroid cancer | class Mollicutes | MR Egger (bootstrap) | 5 | -1.057 | 4.549 | 0.383 | 0.348 | 4.67E-05 | 2.59E+03 | 0.475 |
| Thyroid cancer | class Mollicutes | Weighted median | 5 | 1.376 | 0.797 | 0.084 | 3.959 | 0.831 | 18.866 | 0.137 |
| Thyroid cancer | genus Ruminococcaceae | Inverse variance weighted | 8 | 1.343 | 0.473 | 0.005 | 3.831 | 1.516 | 9.683 | 0.011 |
| Thyroid cancer | genus Ruminococcaceae | Inverse variance weighted (multiplicative random effects) | 8 | 1.343 | 0.452 | 0.003 | 3.831 | 1.581 | 9.283 | 0.009 |
| Thyroid cancer | genus Ruminococcaceae | MR Egger | 8 | 1.875 | 2.436 | 0.471 | 6.518 | 0.055 | 7.73E+02 | 0.530 |
| Thyroid cancer | genus Ruminococcaceae | Simple median | 8 | 1.110 | 0.624 | 0.075 | 3.034 | 0.893 | 10.300 | 0.135 |
| Thyroid cancer | genus Ruminococcaceae | MR Egger (bootstrap) | 8 | -0.176 | 1.604 | 0.445 | 0.839 | 0.036 | 19.440 | 0.517 |
| Thyroid cancer | genus Ruminococcaceae | Weighted median | 8 | 0.710 | 0.654 | 0.278 | 2.033 | 0.564 | 7.328 | 0.385 |
| Thyroid cancer | genus Paraprevotella | Inverse variance weighted | 5 | 1.263 | 0.499 | 0.011 | 3.536 | 1.330 | 9.401 | 0.024 |
| Thyroid cancer | genus Paraprevotella | Inverse variance weighted (multiplicative random effects) | 5 | 1.263 | 0.321 | <0.001 | 3.536 | 1.885 | 6.634 | 4.27E-04 |
| Thyroid cancer | genus Paraprevotella | MR Egger | 5 | 5.760 | 4.782 | 0.315 | 3.17E+02 | 0.027 | 3.73E+06 | 0.420 |
| Thyroid cancer | genus Paraprevotella | Simple median | 5 | 1.372 | 0.667 | 0.040 | 3.941 | 1.067 | 14.561 | 0.079 |
| Thyroid cancer | genus Paraprevotella | MR Egger (bootstrap) | 5 | -0.499 | 2.236 | 0.403 | 0.607 | 0.008 | 48.596 | 0.484 |
| Thyroid cancer | genus Paraprevotella | Weighted median | 5 | 1.211 | 0.612 | 0.048 | 3.356 | 1.011 | 11.140 | 0.091 |
| Thyroid cancer | phylum Actinobacteria | Inverse variance weighted | 12 | -1.389 | 0.370 | <0.001 | 0.249 | 0.121 | 0.515 | 0.001 |
| Thyroid cancer | phylum Actinobacteria | Inverse variance weighted (multiplicative random effects) | 12 | -1.389 | 0.367 | <0.001 | 0.249 | 0.122 | 0.511 | 0.001 |
| Thyroid cancer | phylum Actinobacteria | MR Egger | 12 | -1.908 | 2.637 | 0.486 | 0.148 | 0.001 | 26.076 | 0.530 |
| Thyroid cancer | phylum Actinobacteria | Simple median | 12 | -1.699 | 0.485 | <0.001 | 0.183 | 0.071 | 0.474 | 0.002 |
| Thyroid cancer | phylum Actinobacteria | MR Egger (bootstrap) | 12 | -2.739 | 1.234 | 0.011 | 0.065 | 0.006 | 0.726 | 0.024 |
| Thyroid cancer | phylum Actinobacteria | Weighted median | 12 | -1.793 | 0.499 | <0.001 | 0.166 | 0.063 | 0.443 | 1.18E-03 |
| Thyroid cancer | phylum Tenericutes | Inverse variance weighted | 5 | 1.775 | 0.615 | 0.004 | 5.902 | 1.768 | 19.699 | 1.00E-02 |
| Thyroid cancer | phylum Tenericutes | Inverse variance weighted (multiplicative random effects) | 5 | 1.775 | 0.334 | <0.001 | 5.902 | 3.067 | 11.357 | 1.92E-06 |
| Thyroid cancer | phylum Tenericutes | MR Egger | 5 | -0.417 | 22.924 | 0.987 | 0.659 | 2.02E-20 | 2.15E+19 | 0.987 |
| Thyroid cancer | phylum Tenericutes | Simple median | 5 | 1.420 | 0.816 | 0.082 | 4.137 | 0.836 | 20.469 | 0.137 |
| Thyroid cancer | phylum Tenericutes | MR Egger (bootstrap) | 5 | -1.140 | 4.359 | 0.353 | 0.320 | 6.23E-05 | 1.64E+03 | 0.454 |
| Thyroid cancer | phylum Tenericutes | Weighted median | 5 | 1.376 | 0.824 | 0.095 | 3.959 | 0.788 | 19.890 | 0.142 |
